# Hydrogel Application in Urban Farming: Potentials and Limitations—A Review

**DOI:** 10.3390/polym14132590

**Published:** 2022-06-26

**Authors:** Swarna Devi Palanivelu, Nur Amira Zainul Armir, Amalia Zulkifli, Ainul Hafiza Abdul Hair, Kushairi Mohd Salleh, Keith Lindsey, Muhamad Hafiz Che-Othman, Sarani Zakaria

**Affiliations:** 1 Department of Biological Sciences and Biotechnology, Faculty of Science and Technology, Universiti Kebangsaan Malaysia, Bangi 43600, Selangor, Malaysia; p109377@siswa.ukm.edu.my; 2 Bioresources and Biorefinery Laboratory, Faculty of Science and Technology, Universiti Kebangsaan Malaysia, Bangi 43600, Selangor, Malaysia; amirazainularmir@gmail.com (N.A.Z.A.); amaliaxzulkifli@gmail.com (A.Z.); 3 Centre of Foundation Studies, University Teknologi MARA, Cawangan Selangor, Kampus Dengkil, Dengkil 43800, Selangor, Malaysia; ainulhafiza@uitm.edu.my; 4 School of Industrial Technology, Universiti Sains Malaysia, Gelugor 11800, Pulau Pinang, Malaysia; 5 Department of Biosciences, Durham University, Durham DH1 3LE, UK; keith.lindsey@durham.ac.uk

**Keywords:** agriculture, application, approach, mechanism, modern farming

## Abstract

Urban agriculture plays a vital role in ensuring the self-sufficiency of a great variety of fresh vegetables and nutrients. It promotes a sustainable food system as well as reducing the dependency on imports for the growing population. Urban farming has made it possible for agriculture practices to be implemented anywhere at any time in a sophisticated way. Hydrogel has been introduced in urban agriculture in the past few decades. However, the application of hydrogel in urban agriculture is still being explored in terms of hydrogel types, structure, physical and chemical properties, change due to external factors, and its suitability for different plant species. This review discusses the potentials and limitations of hydrogel in different application conditions. We present the state of knowledge on hydrogel production and crosslinking methods, hydrogel characteristics, water absorption and release mechanisms of hydrogel, hydrogel advantages and limitations, and current and future applications in urban farming.

## 1. Introduction

Urbanisation has led to a reduction in arable land area for the increased population, creating supply problems for food products. The concept of urban agriculture provides solutions for supplying high-quality food products, especially vegetables, and relieves the dependence on traditional farming, which has become more limited. The United Nations Food and Agriculture Organization (FAO) statistics show that fertile land per person is anticipated to decrease to one-third of that available in 1970 (FAO 2016) by 2050 [1,2]. The lack of available land in metropolitan areas creates a need for sustainable agriculture to provide yields at fast and constant rates. However, urban agriculture is limited to a few crops that can be grown under strict growth parameters [3]. Fruits and berries are highly preferred to be grown in urban agriculture, but identifying crop varieties suitable for the constricted growth parameters of the urban agriculture farming system requires customised modifications [3].

Current developments in the urban farming system involve manipulating light, nutrient solution and the plant growth medium. The aspect of water source alone is a major topic of study, as it is essential for plant growth. One of the critical innovations in urban agriculture is the use of hydrogel as one of the main components in the plant growth medium. Hydrogel has been used in agriculture over the past five decades and is efficient as a water-holding reservoir and nutrient mobiliser when used in the soil [4]. Hydrogels constructed of superabsorbent polymers have been used widely in the agriculture industry due to their roles in soil enhancement, allowing plants to grow in arid areas, and facilitating seed germination [5]. 

Hydrogel has been applied to different types of soil in different dosages. Hydrogels’ ability to absorb water 400-times that of their dry weight and release water gradually to reduce the leaching of herbicides and fertilisers improves soil quality and reduces irrigation frequencies, found to be advantageous for agricultural use [6]. A green roof study suggested that a combination of 20% coconut coir and 80% perlite with 1.0 kgm^−3^ hydrogel provided optimum plant growth with enhanced ornamental quality in *Mentha suaveolens* [7]. The growth of lettuce (*Lactuca sativa*) and Chinese mustard (*Brassica juncea var. rugosa*) was tested on 50 g swollen oligoalginate immobilised hydrogel and applied to a deep flow technique in a hydroponic system. The hydrogel of 20% carboxymethyl cellulose (CMC), 20% polyacrylamide (PAM), and oligoalginate sterilised by irradiation at 15 kGy were found to be the best plant growth media compared to coir dust (as a control) [8]. These studies indicate the versatility of hydrogel in urban farming applications.

The types of hydrogels can be classified based on several rationales, such as material origin source, polymeric compositions, configurations, crosslinking types, physical appearances, and electrical network charges. Generally, hydrogels can be prepared from synthetic-based or bio-based materials. Synthetic polymers have petroleum-based products [6,9], while the bio-based hydrogels have natural sources, such as cellulose, starch, lignin, and kenaf fibre [10,11,12,13]. According to Tabata (2009) [14], synthetic-based hydrogels are less hydrophilic and mechanically more robust compared to bio-based hydrogels. However, the bio-based hydrogels are far more preferable to synthetic hydrogels for agriculture application due to their unique advantages, such as non-toxic, biodegradable, low cost, and abundant sources. Cellulose, chitin, and chitosan are examples of natural-based biopolymers with hydrophilic functional groups where they can absorb and retain a large amount of water [5]. Hydrogels can be crosslinked chemically (chemical reactions, polymerisation, graft polymerisation, network formation of water-soluble polymer, and radiation crosslinking) and physically (ionic interactions, hydrogen bonds, hydrophobic interactions). Chemically crosslinked hydrogels have permanent linkages compared to physically crosslinked hydrogels with temporary linkages [15,16].

Even though hydrogels offer great potential in urban agriculture, hydrogel application as a plant growth medium in urban farming is still limited, due to several factors and challenges. Thus, this review article will be focussing on aspects of hydrogel production, the potentials and limitations of hydrogels for greater knowledge about hydrogel behaviour, and hydrogel applications in urban agricultural practices. 

## 2. Hydrogel Production and Crosslinking Methods

The production of synthetic-based hydrogels is traditionally realised through chemical polymerisation methods [16]. As mentioned, bio-based hydrogels are from natural sources and various methods have been employed to obtain bio-based polymers. As an exemplar, fermentation in micro-organisms obtains polyhydroxyalkanoates and the tools of biotechnology are used to obtain poly lactides and poly(butylene succinate) [17]. Bio-based hydrogel derived from cellulose is prepared by cellulose dissolution in an aqueous solvent (NaOH/urea/dH2O solution). The resulting mixture solution is later crosslinked to produce the hydrogel and neutralised in distilled water [18,19]. The first idea of polymer crosslinking was introduced by Charles Goodyear in 1839, aiming for tougher and longer-lasting sulfur-crosslinked rubber properties than uncrosslinked rubber [20]. Crosslinking during hydrogel fabrication creates the three-dimensional polymeric network [21] and prevents the dissolution of polymer chains in a solvent [22]. The concentration of the crosslinker significantly affects the swelling capacity of the hydrogel, whereby a higher concentration of crosslinker will result in lower swelling capacity and vice versa [10,11,12]. Crosslinked hydrogels have numerous enhanced characteristics, such as increased strength, elasticity, and tensile strength [23,24,25]. The characteristics of biocompatibility and biodegradability, and the ability to retain water, have attracted much interest in hydrogel utilisation in various applications. 

**Figure 1:**
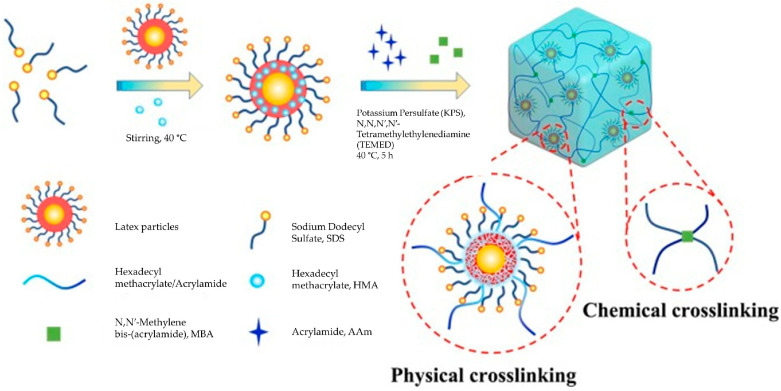
Scheme for synthetic process of hybrid hydrogels. Reproduced from [35] with permission from Elsevier, 2018.

The two major crosslinking techniques (physical and chemical) have been optimised in the construction of hydrogels [26]. A mixture of crosslinking methods has been employed to create novel physicochemical properties for various applications. The significant difference between physically and chemically crosslinked hydrogel is the hydrogel formation via non-covalent and covalent bonds, respectively. A physically crosslinked hydrogel possesses a reversible property that is widely utilised in medical fields, such as drug delivery systems, due to its short life span, sensitivity towards stimuli, self-healing ability (ability to reform after damage), and its non-toxicity, despite being mechanically weak. Examples of the physical crosslinking method are (1) via hydrophobic interaction that employs the hydrophobic and hydrophilic aqueous micellar system and (2) via suspension polymerisation by phase inversion in the dispersed and continuous phases [27,28,29,30]. However, physically crosslinked hydrogels are not typically designated for urban farming applications, given the irreversible and mechanically strong properties of chemically crosslinked hydrogels favoured in urban farming [31,32]. On the other hand, the covalent bonds of chemical hydrogel make it a permanent gel, strong, thermally stable, and controllable, which is ideal for agricultural applications. Therefore, researchers have continuously devised crosslinking methods to obtain improved ways to produce desired hydrogels. For example, the hybrid crosslinking method refers to the use of dual physical and chemical crosslinking techniques [33,34]. This dynamic hybrid crosslinking method exhibits both exceptional chemical and physical crosslinking properties and overcomes weaknesses by forming a strong hydrogel with excellent self-recovery abilities, as shown in Figure 1. The hybrid crosslinked hydrogel in urban farming is able to reform upon stretches of 20-times beyond its initial length [35].

## 3. Hydrogel Characteristics

Hydrogels are constantly being modified for novel properties and applications. The choices of polymers with desired characteristics and crosslinking methods alongside crosslinking degrees are vital steps to produce acceptable qualities of hydrogels for a specific application, especially for agricultural purposes. Fundamentally, according to Flory [36], the swelling of a network dissolved in pure solvent is dependable on the degree of crosslinking and the role of the elastic nature of the polymer chains [31]. The collapse is explained by the temperature being reduced or acetone concentration being increased in the acetone–water gel fluid mixture according to mean-field theory based on a continuation of the work of Flory [37]. Physically, three key factors are considered in measuring the mechanical properties of hydrogel: stiffness, strength, and toughness. Polymer networks generally show low strength and toughness; however, the end applications of hydrogel usually require long-term stability, especially in the controlled or slow-release systems used by urban farming for fertiliser release [38]. Thus, reinforcement is needed, such as composite hydrogels, to maintain structural stability. As an example, a double-network hydrogel has excellent mechanical properties, providing a multi-functional hydrogel [39]. This double-network hydrogel consists of two layers of the polymeric network, where the first layer has highly crosslinked short chains, making it brittle but strong, while partially non-crosslinked long-chain polymers make up the second network, maintaining the hydrogel’s elasticity. The modulus and relaxation performance are advantages of the double-network hydrogel, and its performance is significantly improved compared with a single-network hydrogel, attributable to an enhanced structure of covalent bonding of polyethylene glycol diacrylate (PEGDA) and physical entanglement with a large molecule chain of gellan gum (GG) and also due to the physical entanglement between GG and PEGDA [40]. In another instance, a double network formed with the aid of UV light (365 nm, 50 mW/cm^2^), crosslinked for 5 min, which combines physically crosslinked GG, conferring cell relaxation and chemically crosslinked PEGDA offering elasticity, provides favourable mechanical properties in the system [40]. Compression tests reveal that the thixotropic GG easily ruptures. It is brittle, while PEGDA expresses ductile performance.

The viscoelasticity of hydrogel is very complex as it contains many hydroxyl groups and takes up a large amount of water [41]. Hydrogel’s viscoelasticity can be measured through rheological studies on the amplitude oscillatory shear measurement, with the important parameters being storage modulus (G′), loss modulus (G″), and loss factor (tan δ) [42]. The deformation of energy stored in hydrogel during shear processing defines G′ as the stiffness of hydrogel. In contrast, G″ measures the energy dissipation during the shear process [43]. Tan δ is defined as G″/G′ and, thus, tan δ > 1 or G″ > G′ and tan δ < 1 or G″ < G′, hydrogel behaves as a viscous liquid and elastic solid, respectively [40]. The high-frequency elasticity of hydrogel is formed through the physically crosslinked hydrogel as it can reform upon breakage due to the weak formation of physical bonds, whereas the chemically crosslinked hydrogel has permanent linkages [44]. However, hydrogels with linear and crosslinked polymer composition, the synthetic-based ones with poor biodegradability, may cause toxicity from the crosslinker or synthesising condition while being more durable than the bio-based polymer [16,45,46]. Hydrogels of bio-based polymer composition were observed to have better biodegradability and low toxicity [47,48]. Thus, hydrogels of bio-based polymers are highly suitable for urban farming applications; nevertheless, having more robust and durable hydrogels is a future possibility. 

**Figure 2:**
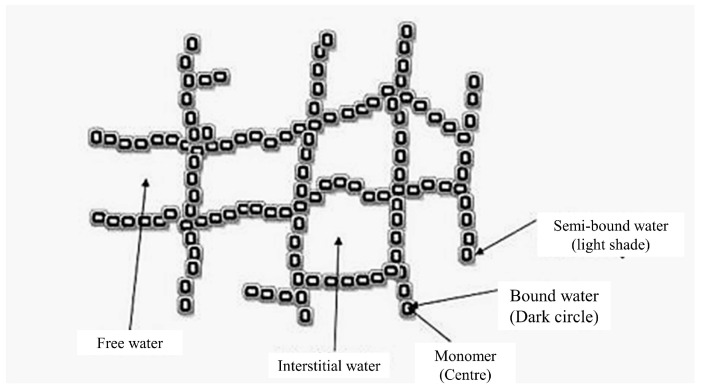
Schematic diagram of molecular structure of hydrogel network with different types of water. Reproduced from [24] with permission from Advanced Pharmaceutical Bulletin, 2017.

Hydrogel is widely used due to its ability and flexibility in water-absorbency properties. These properties help tackle water scarcity, mainly in water-dependent applications, especially in agriculture. With high hydrophilicity, the bio-based polymer-based hydrogel can retain a high volume of water, subject to the crosslinker and polymer types used. In terms of water interaction with hydrogel, there are four types of water associated with hydrogel, namely bound water, free or bulk water, semi-bound water, and interstitial water, as shown in Figure 2 [24]. The combination of primary and secondary water makes the total bound water, whereby the former refers to the first water molecules that enter the hydrogel matrix, dictated by the presence of hydroxyl groups. The latter corresponds to the hydrophobically bound water [24,49]. Upon hydration of the polar group, the hydrated molecules that result in the swelling of polymeric networks will eventually expose the hydrophobic groups in the matrix capable of absorbing water, which hitherto, refers to the secondary water [50]. The osmosis law drives the absorption of additional water, termed as free or bulk water, into the empty spaces between the pores and chains until equilibrium swelling is reached, indicating that a network elasticity is achieved by a crosslinking mechanism [49,50]. Lastly, semi-bound and interstitial water exists between the bound and free water and is physically trapped in the hydrogel polymeric network. Apart from that, the ionic group dissociation during the crosslinking reaction produces additional osmotic pressure when the concurrent increase in counterion numbers inside the hydrogel occurs. This occurrence enables superabsorbent hydrogel to absorb more water compared to ordinary hydrogel [51]. 

## 4. Advantages of Hydrogel in Urban Farming

### 4.1. Hydrogel as a Potting Medium

A potting medium is a growing medium for plants to grow in an optimum condition and for the root system to be established. The potting medium is one of the most important factors influencing plant growth. Even though soil has been commonly used as a potting medium, for which there are various advantages, including its ready availability, there are some disadvantages in its use, such as difficulties in handling and transporting for a large production scale [52], for example, in glasshouses. Conventional farming in soil is prone to soil-borne diseases caused by pathogens and parasites. Soil health is crucial for good plant growth, and the quality of the soil may vary in terms of the microbial community, abundance, diversity, activity, and stability in soil biota as an indication [53]. Furthermore, agricultural soil is also subject to contamination by heavy metals in some industrial areas [54]. Conventional farming is also more costly compared to modern farming approaches, such as hydroponics, in the long run of the operation [55]. Researchers are keen to be free from the trammels of conventional farming using soil, opting for better alternative approaches through modern farming. 

Potting media, such as sphagnum moss, rock wool, cocopeat, and perlite, are lightweight compared to bulky compost [56]. A lightweight growing medium is of significant interest for soilless culture systems due to its ease of handling [57,58,59]. Hydrogel is considered a lightweight material and, therefore, suitable to be used in agriculture [31,60]. Following intense studies on advanced materials, hydrogel is now used as a potting medium [61,62]. The most significant requirement for a potting medium is excellent water-holding features to provide water and nutrients for plant development. Hydrogel was introduced as a potting medium in urban farming in the 1950s and has been used as a seed additive, to improve root establishment of seedlings, and in slow-release coatings [63]. Currently, biodegradable hydrogels have become an exciting field of study in urban farming applications. This is due to the flexibility they offer, through mixing different types of non-toxic cellulose-based materials, such as cellulose derivatives, sawdust cellulose, rice ash, wheat straw, pineapple peel, and oil palm empty fruit bunch, as the precursors in making the hydrogel [45,64,65,66].

Therewithal, Melo and co-workers reported that the growth of tomato seedlings was enhanced by using nanocomposite–calcium montmorillonite (NC-MMt) hydrogel made of PAAm (polyacrylamide compression) and biodegradable polysaccharide CMC (carboxymethyl cellulose) with multiple amendments [66]. In a different study, 0.4% of montmorillonite-embedded carrageenan/psyllium composite hydrogels as soil conditioners increased the water-holding capacity from 0.533 to 0.836 g /g and availability of water content in the soil was above 60% [67]. Moreover, a poly(acrylamide-co-acrylic acid)/silver-coated superabsorbent hydrogel nano-composite demonstrated excellent water-retention capacity, which could be valuable in rainfed agriculture [68]. This aligns with previous studies by de Vasconcelos and co-workers, as the analysis of melon seedling growth was improved due to superabsorbent hydrogel composite contribution [69]. 

### 4.2. Alternative to Soilless Agriculture

Soil is morphologically defined by the Natural Resource Conservation Service (NRCS) as a natural body comprising solids (minerals and organic matter), liquid, and gases that occur on the land surface, occupies space, and is characterised by one or both of the following: horizons or layers that are distinguishable from the initial material as a result of additions, losses, transfers, and transformations of energy and matter or the ability to support rooted plants in a natural environment [70,71]. Soil contributes to plant growth through provisions of a root environment with suitable temperature, pH, microbial community, and root-anchoring features and so provides a support system for plants [72,73,74]. The properties of soil have been studied and then adapted and customised into modern farming technology with more flexibility and convenience, such as nutrient supply and irrigation to plants. Innovation is increasingly necessary, due to the decreasing arable land and growing world population, which demand alternative approaches towards soilless culture in urban farming [2,59]. 

Soilless agriculture is a method of planting crops in any other medium than soil, such as sand, peat, rock wool, sawdust, cocopeat, compost, perlite, vermiculite, zeolite, and volcanic rock [56,59], potentially a practical approach in overcoming water-supply deficiency during plant growth [75]. Hydrogel is in the trend of being used as a potting medium that could be employed to increase the yield of crops and provide nutrients [75,76,77]. Likewise, hydrogel is also considered a soil conditioner, due to its effects on structure and to infiltrate water, minimising soil erosion and water run-off, decreasing soil compaction, supporting plant growth performance when less irrigated, and delaying dissolution of fertilisers to enable nutrient retention [63,69]. Various types of hydrogel could be tailor made to be utilised in soilless agriculture [78]. 

A modus operandi for urban farming was verified in soilless vertical farming based on woven, knit, and non-woven structures integrated with hydrogel as cultivation beds to grow plants, and the application was found to be successful [79]. Recently, 3D printing technology, also known as additive manufacturing, has been expanding in healthcare, automotive, locomotive, and aviation, encompassing agriculture industries [80]. Smart hydrogels embodied with silica-based nanoparticles serve as agrochemical carriers in plants. Additive manufacturing by fabricating hydrogels as receptive structures is suggested for soilless cultivation substrates that would promote plant growth [81]. To summarise, hydrogel has potential as a potting medium in the agricultural sector and could become a suitable soil substitute in soilless agriculture. 

### 4.3. Efficient Irrigation and Fertiliser Application

Water is an essential requirement for plant growth. Thus, a lookout for novel irrigation solutions is likely to become increasingly important in the agriculture sector. Crop plantations in the scarce water environments of arid and semi-arid regions experience adverse effects, as these regions are mainly dependent on rainfall. Conventional agriculture faces issues, such as inefficiency in water use, large land area requirements, high fertiliser loads, and soil degradation [55,82,83]. Hydroponic systems require only 10–15% of the water volumes compared to conventional systems, as shown in Table 1 [55]. 

The water-holding capacity of hydrogel enables water availability for long periods in the soil and the application of hydrogel as a soil amendment can reduce irrigation frequencies [6]. The use of hydrogel in sandy soils has been proven to retain water, thereby decreasing the need to irrigate [56]. In another study, the application of 0.4% hydrogel as a soil amendment improved the water usage efficiency in growing *Agrostis stolonifera* [84]. Hydrogel is highly advocated for growing vegetable crops in agriculture on sandy soils, as shown in hydrogel-treated alluvial and red sandy loam soils. Water was available to plants for more than 1.5–2-times longer compared to non-treated soils [85]. 

On top of that, fertiliser is essential for maximising crop yields. However, excessive use and application of fertiliser to crops can be detrimental to the environment. Over-fertilising can lead to severe problems, such as leaching issues, water eutrophication, soil pollution, and water pollution [86,87]. Generally, nitrogen (N), phosphorus (P), and potassium (K) are considered the primary nutrients for plants to grow. Sources of N are obtained from the atmosphere through biological fixation and fertilisers. P is added to the soil through fertilisers as the naturally available P in soil minerals is insoluble and unavailable to plants readily. K exists naturally in soil minerals but is unavailable to plants, except as soluble soil solutions that are readily available. In conventional agriculture, extensive chemical fertilisers are used to obtain high yields for commercial purposes [82,88]. At present, nanotechnology is of growing interest in plant nutrition in conventional agriculture [89,90]. Nano fertilisers are being introduced, focusing on P fertilisers, as most farmlands are P deficient [91]. 

Hydrogel-based fertilisers have been developed to minimise the frequencies of irrigation and fertiliser application [92]. There are two types of method: slow-release fertiliser (SRF) and controlled-release fertiliser (CRF). SRF is coated physically by hydrophobic materials, which work as a boundary for the fertiliser to release [65]. The rate, pattern, and duration of fertiliser release are not controllable [93]. Meanwhile, CRF is an encapsulated fertiliser within organic or inorganic materials that controls the rate, pattern, and duration of fertiliser release [87]. Producing hydrogels with SRF by loading the urea fertiliser in halloysite nanotubes (HNTs) under vacuum and embedding together with sweet potato starch by free-radical copolymerisation is found to slow down the release rate of fertilizer [92].

Based on previous research by Hendrawan and co-workers, the leaching of fertiliser and deficiency in water supply can be prevented by CRF, as they have manufactured a polyvinyl alcohol (PVA) and *Premna oblongifolia merr* (POM) hydrogel composite, with glutaraldehyde acting as a crosslinker. A zinc nitrate solution was added to the hydrogel mixture to measure the nutrient release [86]. Furthermore, Elbarbary and co-workers prepared hydrogels of various copolymer compositions of polyvinylpyrrolidone (PVP) and carboxymethyl cellulose (CMC), linked by gamma radiation [94]. Alternatively, bio-based and hybrid hydrogels have been developed for slow-release fertiliser purposes [95,96,97]. This formulation and invention resulted in reduced frequencies of fertiliser application while ensuring nutrient sustainability. In Table 2, some of the SRFs and CRFs developed are listed.

Li and co-workers developed a wheat-straw-based semi-interpenetrating polymer network (semi-IPN) hydrogel, incorporating SRF [45]. They found that the particle size, pH, salt solutions, and ionic strength influenced fertiliser release and swelling behaviour. The diffusion coefficients of fertilisers had better swelling capacity in neutral conditions, and the cation and anion effects on swelling go by Na^+^ > K^+^ > Ca^2+^ and Cl^−^ > SO_4_^2−^ order, respectively. An NPK fertiliser-loaded nano-composite showed good slow-release properties, complying with the Committee of European Normalization (CEN) standards, indicating outstanding slow-release properties [45,98]. These fertilisers could be applied in urban farming, where nutrients will be available to the plants for a longer time. According to the fertiliser release mechanism, the fertiliser release rate is expected to be rapid in the first phase. However, as time increases, the release rate of the fertiliser is reduced [101]. On another occasion, Karunaratha and co-researchers [102] described the effectiveness of Fe(III)-alginate hydrogel as an SRF system for tomato plant growth. The hydrogel matrix in the form of a bead with a diameter of 3.5 mm was fabricated by dropping an aqueous alginate solution into 0.1 M FeCl^3^ using an 18-gauge needle at a height of 6 inches. This bead can absorb water due to the hydrogel’s primary characteristic, whereby Fe enables the uptake of nutrients, such as NH^4^, NO^3^, and PO^4^, from a liquid of animal waste. The nutrient release assessment from the hydrogel was tested on the growth progress of tomato (*Solanum lycopersicum*) plants compared with conventional fertiliser with the same nutrient content. The fertilised bead promoted fruit and root growth in tomatoes, comparable with the conventional fertiliser solution.

On top of that, the use of hydrogel as a nutrient immobiliser or reservoir for vegetable growth in urban farming was discussed by Luan and Xo (2017) [8]. Their hydrogel was prepared by grafting crosslinked PAM chains onto CMC using γ-irradiation with the addition of olioalginate and nutrients. The addition of oligoalginate in the formulation and moisture control in the medium by hydrogel enhanced the seed germination. Furthermore, alginate is found to help in slow release through the alginate lyases, which are involved in the biosynthesis of alginate [103,104]. The hydrogel with added nutrients and supplement was tested in a hydroponic system for planting seeds of Chinese mustard (*Brassica juncea*) and lettuce (*Lactuca sativa*). The seeds of both plants were planted directly in the hydrogel and seedling growth was much better in terms of root length, shoot height, and total fresh biomass than plants planted on coir dust. However, the hydrogel started to collapse in the third week and completely collapsed in the fifth week but still supported plant growth prior to the harvesting period [8]. As a complement, Tempo-oxidised bacterial cellulose (TOBC)-sodium alginate (SA) hydrogel composites were found to have better compression strength and chemical stability. TOBC is reported to substantiate the structural, mechanical, and chemical stability of the composites [105,106]. In a separate study, hydrogels were observed for adsorption of contaminants in water and wastewater treatment. The hydrogel was fabricated in two steps with the adsorption of rhodamine B in an aqueous solution for improved stability and swelling ratio and chitosan interacted with polyacrylonitrile (PAN) to form chitosan/PAN films and then crosslinked with epichlorohydrin (ECH) to make hydrogel films. The addition of PAN was found to enhance the stability of the fabricated hydrogel film [107,108].

Hydrogel application in fruit and vegetable planting with said examples decreases water usage, reduces the watering frequency, and locks the soil nutrients to sustain plant growth. Fruit crops, such as melon, citrus, and strawberry, are suitable for urban farming [60]. The application of hydrogels for fruit plant growth has been extensively investigated. de Vasconcelos and co-workers developed a starch-grafted-poly (sodium acrylate) matrix, a superabsorbent hydrogel composite filled with husk rice ash (RHA) to overcome osmotic stress in vine fruit plants, such as melon (*Cucumis melo*). The superabsorbent hydrogel/rice hush ash composite was tested for two different conditions. In the first analysis, different types of the substrate with hydrogel (0, 0.5, 1.5 or 2.0 *w/w*%) were tested, seed was germinated till the 4th day, and after the eighth day, irrigation ceased and growth was monitored till the 19th day. In the second analysis, different types of soil with hydrogel (0, 0.25, 0.5, 0.75 and 1.0 *w/w*%) were tested, seed was germinated till the 4th day, and after the 8th day, irrigation ceased and the experiment was observed till the 25th day. In the first experiment, plant growth was the highest (5.60 cm) for coconut fibre substrates as compared to sandy soil (4.12 cm) substrates. The presence of hydrogel only impacted the plant roots’ size. In the second experiment, clay soil treated with hydrogel led to 10% greater seedling height except for 1% hydrogel compared to the non-conditioned soils [69]. Numerous studies reporting on the application of hydrogel for leafy vegetable growth [60,102,109] studied choy sum, a leafy vegetable using superabsorbent hydrogel derived from a soy product’s manufacturing waste, called okara, as a soil supplement. The okara-poly (acrylic acid-co-acrylamide) superabsorbent hydrogels were synthesised through a grafting copolymerisation of monomers on fresh okara crosslinked with N, N′-methyl- enebisacrylamide. The yield of vegetables grown in substrates supplemented with 3% (*w/w*) increased by 88%, and substrates supplemented with 5% (*w/w*) of gel increased by 113%, compared to the control (no hydrogel). Okara-poly(acrylic acid) (Ok− PAA) polymers too were produced to be reused in agriculture [110]. Hydrogels from diapers have been sterilised and recycled into cylindrical turbines. These hydrogels planted near the lateral roots of trees maintained moisture around the root [111]. As discussed above, biomass agricultural waste was turned into nanocellulose-based superabsorbent hydrogels, significantly decreasing frequencies of irrigation and fertiliser application [112].

## 5. Limitations of Hydrogel in Urban Farming

### Structural Stability and Physical Integrity of Hydrogel

Despite the clear benefits of hydrogel in urban farming, there are also limitations. The structural and physical properties of a hydrogel are the backbones of hydrogel formation. As the use of hydrogel broadens, each application faces challenges to obtain an optimum form of hydrogel that suits a specific use. One of the significant limitations of hydrogel is the low mechanical strength and fragility in a hydrogel that could be a major problem to sustain [46]. While synthetic-based hydrogels, such as acrylic acid and polyacrylamide, are more difficult to degrade in the soil, their heavy use in the soil is not advised as they may release biologically toxic residues in the environment [113]. 

Bio-based hydrogels are mechanically weak compared to synthetic-based hydrogels, making them more biodegradable, leading to easy degradation [47]. Thus, hydrogels would need frequent replacement as a consequence of their fleeting degradability nature. These hydrogels are made biodegradable to reduce environmental hazards; however, they are short lived. Bio-based hydrogels, such as from lignocellulosic materials, also face problems of cellulose dissolution. Cellulose is difficult to dissolve in water and common solvents [19,114]. Producing this ‘smart-material’ hydrogel involves solvent, crosslinkers, and human sources, which are costly [10]. Therefore, a more economical and practical bio-based hydrogel for agriculture application is needed.

In addition, the carbon material functional groups in hydrogel will form hydrogen bonds with water molecules that could give rise to water sorption with organic solutes in a competitive manner [115], which could potentially create an imbalanced fulcrum in transporting water. In a recent study, potato-starch-based hydrogel was used as a seed coating to improve the early growth of corn seeds. Only a slight improvement was observed in water absorption by the hydrogel when water was supplied at 65%, 82%, and 92% field capacity. There was no significant difference in the dry weights of the coated and uncoated corn plant roots and shoots after 14 and 21 days of growth, suggesting hydrogel coating is not impactful [116]. Furthermore, a study reported three species of ornamental plants, *Gazania rigens, Pelargonium peltatum*, and *P. zonale*, which were grown in pots with a peat-based substrate, tested with the presence of commercial inoculum of arbuscular mycorrhizal fungi (AMF) and hydrogel. The hydrogel led to a decreased diameter for the largest flower of *G. rigens*, decreased flowering of *P. peltatum*, and negatively affected *P. zonale* plants. It was concluded that the inclusion of hydrogel in the potting medium had no positive effects on any of the parameters of the three tested species of plants, as shown in Table 3 [117]. The hydrogel underperformance could be due to several reasons. It may be that the hydrogel effect is transient and the positive effects of the gel on soil might be observed only for a short time. Furthermore, frequent dehydration and hydration routines in the hydrogel may have slowed down the positive effect on the water-retention qualities. It is known that the concentration of gel is essential for the hydrogel to deliver a positive impact on the soil [117]. 

## 6. Current and Future Applications of Hydrogel in Urban Farming

Hydrogel characteristics of biocompatibility, environmental friendliness, and capacity to absorb water are the basic principles governing their extensive range of use in urban farming. Conversely, the poor structure of hydrogels, due to low mechanical strength and toxic crosslinkers, represents drawbacks. Nevertheless, the literature shows that the development, production, and application of new hydrogels may overcome these difficulties. Another major concern in urban farming is the plant’s susceptibility to pests and pathogens, adversely affecting plant growth and decreasing productivity. López-Velázquez et al. [47] succeeded in developing a hydrogel that can reduce *Phytophthora capsica* attacks in chilli plants. This pathogen causes a disease known as chilli wilt, which delays plant growth, causes wilting and black colouration in the roots, stem, and fruits, consequently decreasing yield (Callaghan et al., 2016) [118]. The hydrogel was formulated by mixing chitosan, gelatin, and polyvinyl alcohol (PVA) in its matrix, followed by the addition of inulin isolated from Dahlia tubers. Inulin is a fructan that can activate an innate receptor-mediated immune response, hence, protecting the plant from further disease symptoms [119]. The efficiency of the inulin/CS/Gel/PVA-dried hydrogel powder was assessed by placing it near the root area, and 80% of the plants survived when treated with inulin-loaded hydrogel without any symptom of the chilli wilt disease. However, plant height was reduced compared to the control sample.

Nevertheless, inulin-loaded hydrogels have good potential to be used as a carrier or immobiliser for pesticides, thus, reducing pathogen infection risk during plant growth. Ma et al. [120] also reported hydrogel fabrication that can minimise pathogen attack towards plant growth. A microsphere hydrogel was prepared in their study by loading the essential oil citral into the copolymer hydrogel matrix, consisting of chitosan and carboxymethyl cellulose. Citral was fixed into the microsphere hydrogel matrix by coupling the amino group of chitosan with the carbonyl group of citral to form a Schiff base structure. According to Adukwu et al. [121], citral has exhibited anti-fungal and antibacterial properties against a wide range of pathogens. The microsphere hydrogel efficiency was evaluated in tomato plants contaminated with pathogenic fungus, *Botrytis cinerea*. The hydrogel matrix also offers antibacterial activity, as it can inhibit the growth of both Gram-positive and -negative bacteria, such as *Escherichia coli*, *Staphylococcus aureus*, and *Bacillus subtilis*.

## 7. Successful Applications of Hydrogel-Based Products

**Figure 3:**
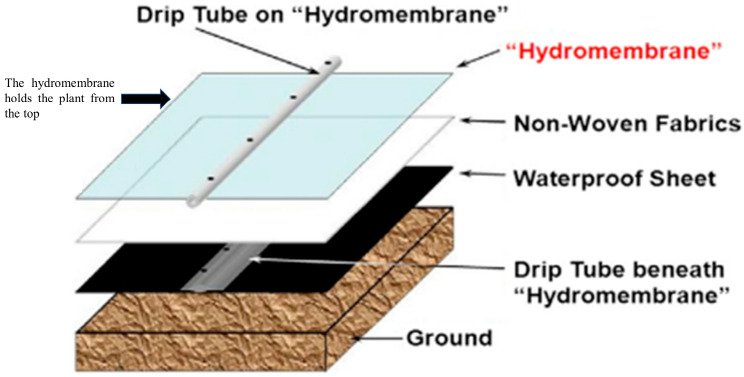
Core elements of Imec system. Reproduced from [122] with permission from Elsevier, 2012.

One of the challenges in urban farming is the fast pace of science and technology, leading to competition. Any new invention creates competition among the existing product in terms of efficacy, benefits, and cost. Urban farming is evolving and discoveries are being welcomed due to their clear potential. For example, film farming can be pointed out as a competitor to hydrogel in urban farming. Film farming was developed by Mori (2013) [122], displayed in Figure 3, which is a combination of hydrogel and membrane technology, known as “hydromembrane”, under the brand name Imec. This hydromembrane is a thin film that can hold plants while filtering viruses and microbes, protecting plants from diseases. This film is also a waterproof sheet that eliminates water run-off and minimises contamination [77]. Imec has been used in cultivating tomatoes, cucumber, melon, paprika, and lettuce. Film farming usually provides a significantly lower yield, but with a much better taste [77]. Regarding the cost, a merge of film farming and vertical farming is regarded as sensible [122]. Thus, this represents a competitive technology for hydrogel.

## 8. Conclusions

This review highlights the practicability of hydrogel in urban farming. Hydrogels may aid agriculture, but they come with constraints in terms of sustainability, cost, and application. Synthetic-based hydrogels are better compared to bio-based hydrogels in terms of material and application in other industries. In agriculture, bio-based hydrogels are favoured over synthetic-based hydrogels for their eco-friendliness; however, this is subject to limitations, so that bio-based hydrogels are still not widely used in this industry. Hydrogels are not suitable for growing all types of plants. As compared to traditional methods, hydrogel does not provide outstanding results in terms of plant yield. Currently, the outcome of plants grown in the presence of hydrogel is not promising, but the technology may evolve as an addition to existing modern farming techniques. By understanding and addressing the challenges, we should be able to address the shortcomings of hydrogel application in agriculture. Hydrogel application in agriculture requires more profound studies with a deep understanding of the interdependence between hydrogel material and plant physiology, though significant progress has been achieved in the fundamental science of polymeric gels and hydrogels [123,124].

## Figures and Tables

**Table 1:** Percentage of water and fertilizer consumption, vegetable yield percentage, and the percentage of water productivity for different new farming systems as compared with the conventional farming system. Reproduced from [55] with permission from American Scientific Research Journal for Engineering, Technology, and Sciences (ASRJETS), 2017.

Parameters	Hydroponic System					
	Media Soilless System		Nutrient Solution System		Aeroponics	Aquaponics
	Open	Closed	Open	Closed		
% Irrigation water saving	80	85	85	90	95	%85–80
% Fertilizer saving	55	80	68	85	85	%99–85
% Productivity increase	100	150	200	250	300	%150–100
% Water productivity	1000	1600	2000	3500	8000	1000–1600

**Table 2:** Examples of slow-release and controlled-release fertilisers developed.

Type of Fertiliser	Composition	Crosslinker	Technique	Rate of Release	Reference
Slow-Release	Sulfonated-carboxymethyl cellulose/ polyvinylpyrrolidone/silica/NPK	N,N′-methylene bisacrylamide (MBA)	In-situ graft polymerisation	Excellent slow-release properties	[98]
	Sawdust cellulose- polyacrylic acid monomer (PAA)/ polyacrylamide(PAM)/Urea	N,N′-methylenebis acrylamide (MBA)	Graft Copolymerization	210 g/g was the best, suitable	[99]
	Cocopeat/Poly Acrylic Acid/NPK	N,N′-methylene bisacrylamide (MBA)	In-situ graft polymerization	Slow release property is better than the commercial super absorbance polymer (CSAP)	[100]
Controlled Release	Starch-g-(acrylic acid-co-methyl methacrylate), carbendazim-loaded hydrogels (CLHs)	N,N′-methylene bisacrylamide (MBA)	Polymerization	Good performance	[13]
	Polyvinylpyrrolidone/ carboxylmethyl cellulose	-	Gamma radiation	Treatment of slow-release fertiliser shows better performance than the untreated soil	[94]
	κ-carrageenan-based hydrogel	Glycerol	Chemical Crosslinking	It may have potentials as a control released fertiliser	[87]

**Table 3:** Effects of factors (1—watering, 2—arbuscular mycorrhizal fungi (AMF) inoculation, 3—hydrogel) and their interactions on various growth parameters and mycorrhizal colonization of roots of model plants according to three-way ANOVA (presented as F value and significance) or according to generalized linear model for number of branches (presented as Wald statistics and significance): * *p* < 0.05, ** *p* < 0.01, *** *p* < 0.001; ns—no significant effect. Arrows indicate positive (↗) or negative (↘) effect, if clearly identifiable. Reproduced from [117] with permission from Elsevier, 2019.

Plant	Factor	Number of Flowers	Diameter of Largest Flower	Shoot Dry Weight	Root Dry Weight	Plant Height/Total Length of Branches (*P. peltatum*)	Number of Branches	Total Leaf Area	Shoot P Concentration	Mycorrhizal Colonization
*Gazania rigens*	(1) watering	20.2 *** ↘	ns	5.5 ** ↘	ns	4.5 * ↘	—	8.2 *** ↘	5.6 ** ↘	18.9 *** ↗
	(2) AMF	31.1 *** ↗	86.5 *** ↗	20.0 *** ↗	ns	76.5 *** ↗	—	40.5 *** ↗	13.8 *** ↗	—
	(3) gel	ns	4.9 * ↘	ns	ns	ns	—	ns	ns	ns
	1 × 2	ns	3.4 *	6.7 **	ns	14.2 ***	—	5.5 **	ns	—
	1 × 3	ns	ns	ns	ns	ns	—	ns	ns	ns
	2 × 3	ns	ns	ns	ns	ns	—	ns	5.7 *	—
	1 × 2 × 3	ns	ns	ns	ns	ns	—	ns	ns	—
*Pelargonium peltatum*	(1) watering	6.6 ** ↗	—	ns	ns	4.6 *	ns	7.0 ** ↗	3.8 * ↘	42.5 *** ↗
	(2) AMF	ns	—	6.2 * ↗	ns	11.4 ** ↗	ns	25.0 *** ↗	14.5 *** ↗	—
	(3) gel	6.7 * ↘	—	ns	ns	ns	ns	ns	ns	10.4 ** ↘
	1 × 2	3.3 *	—	ns	ns	ns	ns	3.9 *	ns	—
	1 × 3	ns	—	ns	ns	ns	ns	ns	ns	ns
	2 × 3	ns	—	ns	ns	6.8 *	ns	14.2 ***	ns	—
	1 × 2 × 3	ns	—	ns	ns	ns	ns	ns	ns	—
*Pelargonium zonale*	(1) watering	ns	—	11.5 *** ↘	24.4 *** ↘	13.4 ***	ns	26.8 ***	4.4 * ↗	12.2 *** ↗
	(2) AMF	148.5 *** ↗	—	169.9 *** ↗	190.4 *** ↗	378.0 *** ↗	62.4 *** ↗	442.5 *** ↗	52.5 *** ↗	—
	(3) gel	7.3 ** ↘	—	ns	ns	ns	ns	ns	ns	ns
	1 × 2	ns	—	ns	7.5 ***	9.3 ***	ns	12.1 ***	ns	—
	1 × 3	ns	—	ns	ns	ns	ns	5.6 **	3.6 *	8.7 ***
	2 × 3	ns	—	ns	ns	6.5 *	ns	ns	7.9 **	—
	1 × 2 × 3	ns	—	ns	4.4 *	ns	ns	ns	ns	—

## Data Availability

The data presented in this study are available on request from the corresponding author.
